# Burden and Outcomes of Gastrointestinal Complications After Kidney Transplantation: A Retrospective Event-Based Study

**DOI:** 10.7759/cureus.109972

**Published:** 2026-05-31

**Authors:** Mohamed Yassir Tahri, Selim Benhadda, Loubna Benamar, Naima Ouzeddoun, Amal Zniber

**Affiliations:** 1 Nephrology, Centre Hospitalo-Universitaire Ibn Sina, Rabat, MAR

**Keywords:** acute kidney injury, deterioration of kidney graft function, global graft survival, infectious diarrhea, kidney transplantation, live donor kidneys

## Abstract

Introduction

Gastrointestinal complications (GICs) are common after kidney transplantation (KT) and may compromise graft function. Our study aimed to determine the prevalence of acute GICs, identify the associated risk factors, and evaluate their impact on graft survival.

Methods

We conducted a retrospective study including patients who experienced an acute GIC defined as any acute digestive event lasting less than 14 days after KT. We included infectious or drug-induced diarrhea, gastrointestinal bleeding, and surgical abdominal complications. Patients with chronic gastrointestinal or hepatic diseases were excluded. As multiple GICs could occur in the same patient, the analysis was performed at the event level. A logistic regression analysis was conducted to identify factors associated with graft dysfunction, defined as an estimated glomerular filtration rate (eGFR) < 60 mL/min/1.73 m² at three months following a GIC.

Results

Fifty-three patients experienced at least one GIC, corresponding to a prevalence of 46.9%, with a total of 84 events recorded. The mean age was 50 ± 13.3 years, with a male-to-female ratio of 1.52. The mean time to onset was 34.5 ± 46.1 months. Acute diarrhea was the most frequent complication (85.71%), which was of infectious origin in 79% and drug-related in 21%. Hemorrhagic complications accounted for 10%, including five cases of hematemesis and four cases of rectal bleeding. Three surgical complications were identified: appendicitis, colonic obstruction, and cholecystitis. From a renal perspective, 47% of patients developed acute kidney injury (AKI), and 20% progressed to graft dysfunction. The occurrence of AKI had a significant impact on six-year graft survival (p = 0.002). In univariate analysis, AKI (OR = 18.75, 95% CI = 3.60-97.5, p < 0.001) and recurrent diarrhea (OR = 2.91, 95% CI = 1.29-6.55, p = 0.010) were significantly associated with graft dysfunction. In multivariate analysis, both AKI (adjusted OR = 41.69, 95% CI = 4.75-365.82, p < 0.001) and recurrent diarrhea (adjusted OR = 3.62, 95% CI = 1.01-13.04, p = 0.049) remained independent predictors of graft dysfunction following GIC episodes.

Conclusion

Infectious diarrhea is the most common GIC after KT. AKI is the main determinant of graft dysfunction and graft survival.

## Introduction

Kidney transplantation (KT) is considered the gold standard treatment for patients with end-stage renal disease (ESRD), as it confers a significant survival advantage and substantial improvement in quality of life across all age groups [1]. However, the success of transplantation largely depends on achieving a careful balance between preventing graft rejection and minimizing complications [2].

Gastrointestinal complications (GICs) are among the most common adverse events in KT recipients, affecting approximately 40% of patients [3]. It arises from multiple etiological factors. Immunosuppressive agents, including calcineurin inhibitors, mTOR inhibitors, corticosteroids, and mycophenolate mofetil (MMF), are major contributors due to their gastrointestinal toxicity. Additionally, infectious causes remain highly prevalent in this population and play a key role in post-transplant morbidity [4-8].

From a prognostic perspective, these complications may lead to significant morbidity, prolonged hospitalization, and impairment of graft function. This highlights the importance of appropriate monitoring and close multidisciplinary collaboration between nephrologists, gastroenterologists, and infectious disease specialists.

The aim of this study was to determine the prevalence and etiological profile of GICs following KT. We also aimed to identify predictive factors associated with chronic graft dysfunction and to assess the impact of these complications on graft survival.

## Materials and methods

This monocentric retrospective study was conducted in the Kidney Transplantation Unit of the Nephrology Department at Ibn Sina University Hospital in Rabat, including 113 renal transplants and covering the period from January 1998 to December 2024. Data collection included demographic, clinical, biological, and microbiological variables.

Following transplantation, patients received antimicrobial prophylaxis with trimethoprim-sulfamethoxazole (TMP/SMX), initiated on postoperative day two or three and continued for six months to prevent *Pneumocystis jirovecii* pneumonia. *Cytomegalovirus* (CMV) prophylaxis was tailored according to donor-recipient serostatus; however, due to limited availability of valganciclovir, acyclovir was used as an alternative for a duration of six months post-transplantation.

GICs were defined as any acute gastrointestinal event occurring after KT, with a duration of ≤14 days and requiring medical, endoscopic, or surgical management. Patients presenting with chronic gastrointestinal disorders (duration >14 days) and/or hepatic involvement were excluded. Chronic gastrointestinal disorders include pre-existing conditions such as inflammatory bowel disease (Crohn's disease and ulcerative colitis), celiac disease, chronic viral hepatitis (hepatitis B and C), liver cirrhosis of any etiology, and any other chronic hepatic or intestinal pathology documented prior to the event.

These complications were subsequently classified into three main categories: acute diarrhea, gastrointestinal bleeding, and surgical abdominal complications.

Acute diarrhea was defined as the occurrence of at least three loose or liquid stools per day, or a significant increase in stool frequency compared to baseline, irrespective of the underlying etiology. In order to further characterize diarrheal events, all patients underwent a standardized diagnostic workup, including stool analyses to identify infectious causes such as bacterial, parasitic, and viral pathogens. CMV infection was confirmed by quantitative PCR and, when indicated, by colonic biopsies demonstrating histopathological evidence of CMV involvement. In the absence of an identifiable infectious cause, drug-induced diarrhea was considered. MMF-related toxicity was supported by characteristic histological findings on colonic biopsies, whereas tacrolimus-associated diarrhea was retained as a diagnosis of exclusion after ruling out all other potential etiologies. Recurrent diarrhea was defined as the occurrence of two or more distinct diarrheal episodes during the follow-up period, with each episode separated by an asymptomatic interval of at least four weeks.

Beyond diarrheal manifestations, other GICs were also assessed within the same framework. Gastrointestinal bleeding was defined as any hemorrhage arising from the upper or lower gastrointestinal tract, while surgical abdominal complications were identified when clinical findings suggested an acute abdomen, including abdominal guarding or rigidity, along with cessation of stool and gas passage for at least 48 hours.

During follow-up, all GICs were systematically recorded for each patient. Given that multiple events could occur in the same individual, the analysis was performed using an event-based approach.

To better understand their temporal distribution, GICs were further stratified according to the time of onset into three phases: early (<6 months), intermediate (6-12 months), and late (>12 months) following transplantation.

Finally, the impact of GIC on graft function was evaluated. For each event, the occurrence of acute kidney injury (AKI) and/or chronic graft dysfunction was analyzed. AKI was defined according to the Kidney Disease: Improving Global Outcomes (KDIGO) criteria [9] as an increase in serum creatinine ≥3 mg/L within 48 hours, an increase to ≥1.5 times baseline, or a urine output <0.5 mL/kg/h for at least six hours, while chronic graft dysfunction was defined as an estimated glomerular filtration rate (eGFR) <60 mL/min/1.73 m² assessed after the gastrointestinal event. Patients with pre-existing graft dysfunction prior to the GIC episode were excluded from this analysis.

Data entry and statistical analyses were performed using JAMOVI software (version 2.5.5). Qualitative variables were expressed as proportions and percentages, while quantitative variables were presented as means ± standard deviation or medians with interquartile ranges, depending on data distribution.

Comparisons between two groups were performed using the Mann-Whitney U test for continuous variables and the chi-square test or Fisher's exact test for categorical variables, as appropriate. Subsequently, the association between explanatory variables and graft dysfunction was assessed using univariate and multivariate analysis based on binary logistic regression. Results were expressed as odds ratios (ORs) with their corresponding 95% confidence intervals (95% CI). To avoid clustering effects, statistical analyses were performed at the patient level. Each patient was counted once, regardless of the number of GIC episodes experienced, based on whether graft dysfunction occurred following the event.

Graft survival was estimated using the Kaplan-Meier method and compared between groups using the log-rank test. Survival time was defined as the interval between transplantation and the occurrence of graft dysfunction or the date of last follow-up. Data were censored for patients who did not experience the event during the follow-up period. A p-value of less than 0.05 was considered statistically significant.

## Results

In our study, GICs occurred in 46.9% of kidney transplant recipients, with 53 patients experiencing at least one event and accounting for a total of 84 episodes. The mean age of the patients was 50 ± 13.3 years (range = 21-73), with a male-to-female ratio of 1.52. The majority of patients (57%) had an undefined etiology of ESRD. Most patients (98%) received a kidney from a living related donor, with donor-recipient human leukocyte antigen (HLA) compatibility detailed in Table 1.

**Table 1:** Patients characteristics. Data are presented as percentages (%) or mean ± SD. CMV: Cytomegalovirus; HLA: human leukocyte antigen; ATG: anti-thymocyte globulin.

Characteristics	Overall (n = 53)
Age (years)	50 ± 13.3
Male sex	32
Sex ratio (M/F)	1.52
Age at transplantation (years)	33 ± 13.9
Living donor	52 (98.1)
HLA matching	
≤2 matches	19 (35.8)
3-4 matches	23 (43.4)
≥5 matches	11 (20.8)
CMV status	
D+R+	50 (94.3)
D−R+	2 (3.8)
D+R−	1 (1.9)
D−R−	0 (0)
Induction therapy	
ATG	39 (73.6)
Basiliximab	6 (11.3)
Methylprednisolone alone	8 (15)
Maintenance therapy	
Tacrolimus	35 (66.0)
Cyclosporine	18 (34.0)
Mycophenolate mofetil	50 (94.3)
Mycophenolate sodium	2 (3.8)
Azathioprine	1 (1.9)
Medical history	
Hypertension	20 (37.7)
Diabetes	2 (3.8)
Heart disease	2 (3.8)
Helicobacter pylori gastritis	17 (32.1)
Gastric ulcer	6 (11.3)
Tuberculosis	6 (11.3)
Smoking	3 (5.7)

Immunosuppressive therapy was primarily based on anti-thymocyte globulin (ATG) for induction (73.6%), followed by basiliximab (11.3%), while maintenance regimens predominantly included MMF (94.3%), tacrolimus (66%), and corticosteroids, with less frequent use of cyclosporine (34%) and azathioprine (1.9%).

The mean number of complications per patient was 1.54, and the mean time to onset was 34.5 ± 46.1 months.

**Figure 1:**
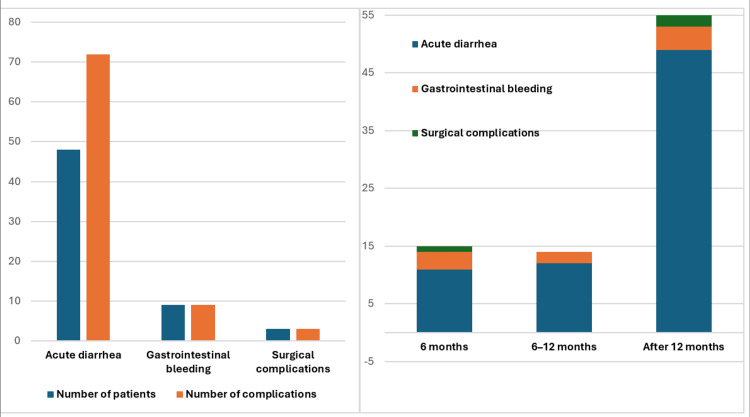
Type and temporal distribution of gastrointestinal complications following kidney transplantation. The left panel shows the distribution of patients (n = 53) and gastrointestinal complication events (n = 84) according to complication type. The right panel illustrates the temporal distribution of acute gastrointestinal complications after transplantation (<6 months, 6-12 months, and >12 months). Data are presented as the number of patients and the number of events.

Acute diarrhea was the most common manifestation, affecting 41 patients (77.35%) and accounting for 72 episodes (85.71%), highlighting a high recurrence rate. In contrast, hemorrhagic complications were less frequent (16.98%), and surgical complications remained rare, observed in only three patients (5.66%) (Figure 1).

Analysis of the timing of occurrence showed a clear predominance of late-onset events, with most complications developing beyond 12 months after transplantation, largely driven by diarrheal episodes. In contrast, early complications, occurring within the first year post transplant, were less frequent and were also mainly represented by diarrhea, while gastrointestinal bleeding and surgical complications remained uncommon (Figure 1).

**Figure 2:**
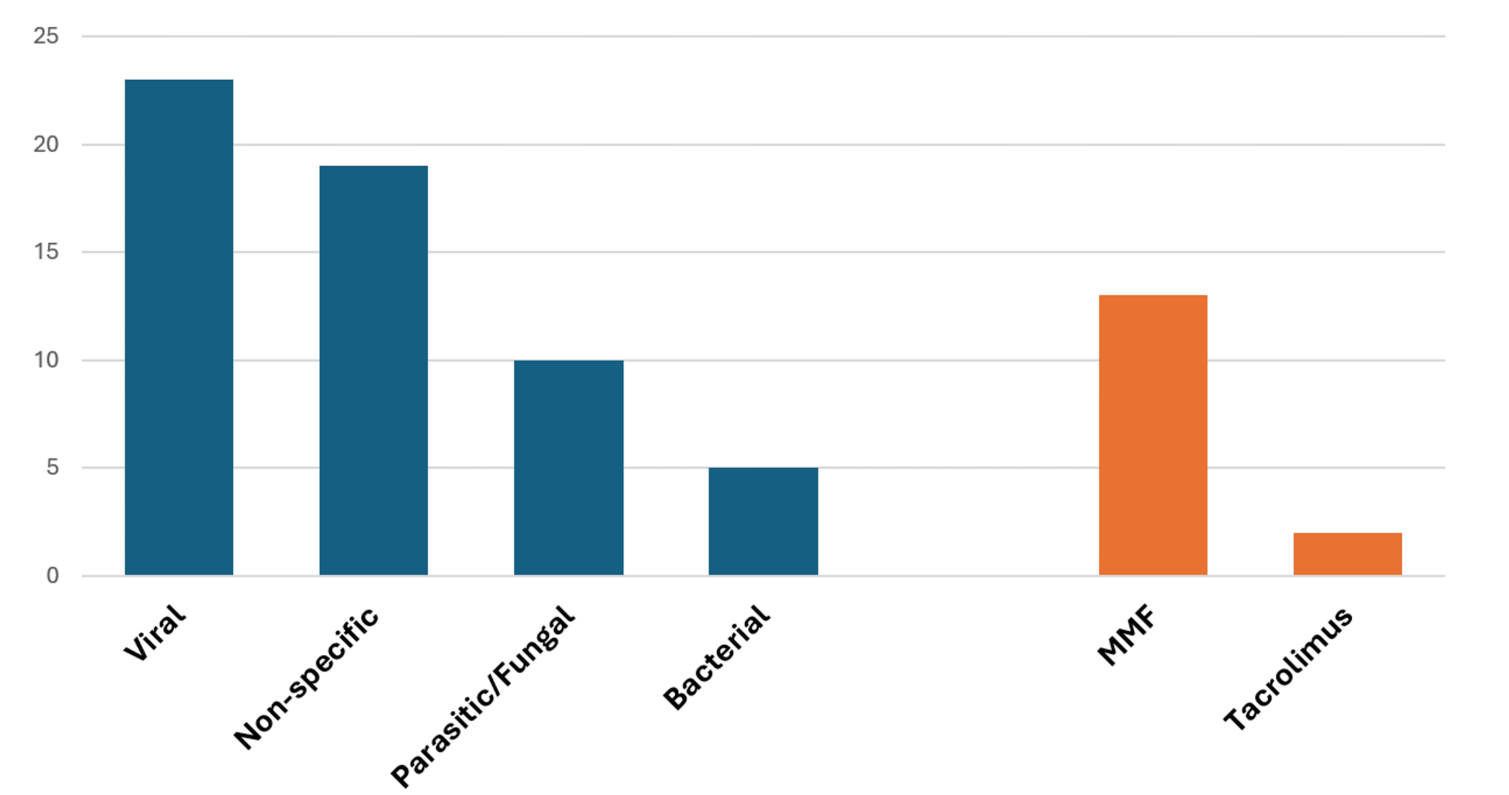
Infectious and drug-related etiologies of acute diarrhea in kidney transplant recipients. Data are presented as the number of diarrhea episodes (n = 72). MMF: mycophenolate mofetil.

In our series, acute diarrhea was predominantly of infectious origin (≈79%), with drug-related causes accounting for a minority (≈20%). Viral infections were the leading etiology (Figure 2), notably COVID-19 (19.44%), followed by CMV colitis (11.11%) and rotavirus infection (1.38%). Bacterial causes were less frequent, mainly involving *Clostridium* species (4.16%), with isolated cases of *Shigella* infection and cecal tuberculosis (1.38% each). Fungal and parasitic infections were also identified, predominantly *Candida albicans* (6.94%), followed by cryptosporidiosis and *Entamoeba* infection (2.77% each), and a single case of cryptococcosis (1.38%). A substantial proportion of cases were attributed to systemic infections associated with diarrhea, including clinically diagnosed gastroenteritis (12.5%), acute pyelonephritis (9.72%), septicemia (2.77%), and diverticulitis (1.38%) (Table 2). Regarding drug-related etiologies, MMF was the most frequently implicated agent (n = 13), while tacrolimus was only rarely involved (n = 2) (Figure 2).

**Table 2:** Infectious etiologies of acute diarrhea in kidney transplant recipients. Data are presented as the number of diarrhea episodes (n = 72). Data are presented as percentages (%).

	n = 72 (%)
Bacterial	
Clostridium	3 (4.16)
Shigella	1 (1.38)
Cecal tuberculosis	1 (1.38)
Viral	
COVID-19	14 (19.44)
Cytomegalovirus	8 (11.11)
Rotavirus	1 (1.38)
Fungal/Parasitic	
Candida albicans	5 (6.94)
Cryptosporidiosis	2 (2.77)
Entamoeba	2 (2.77)
Cryptococcosis	1 (1.38)
Systemic infections with associated diarrhea	
Gastroenteritis	9 (12.5)
Acute pyelonephritis	7 (9.72)
Septicemia	2 (2.77)
Diverticulitis	1 (1.38)

Gastrointestinal bleeding represented the second most frequent category of GICs (n = 9), with hematemesis as the predominant presentation (approximately two-thirds of cases), while rectal bleeding accounted for the remaining third of cases. The main underlying cause was gastric ulcer disease, followed by internal hemorrhoids, with anal fissures and cecal ulcers observed at similar frequencies, and esophagitis reported less commonly. Abdominal surgical complications were rare, occurring in only three patients, and included colonic obstruction, acute appendicitis, and cholecystitis.

Overall, GIC had a significant impact on graft function, with AKI occurring in nearly half of the cases (47%). Renal function recovered to baseline in the majority of patients (80%), whereas 20% developed persistent graft dysfunction, underscoring the potential severity of these events.

Comparison between patients who developed chronic graft dysfunction and those who recovered baseline renal function after GICs identified several associated factors.

In univariate analysis, several variables were associated with graft dysfunction following GIC episodes. The occurrence of AKI was the strongest predictor of graft dysfunction, with an odds ratio of 18.75 (95% CI = 3.60-97.5, p < 0.001). Recurrent diarrhea was also significantly associated with graft dysfunction (OR = 2.91, 95% CI = 1.29-6.55, p = 0.010). Drug-induced acute diarrhea showed a trend toward significance (OR = 6.35, 95% CI = 0.84-48.0, p = 0.070), as did age (OR = 1.04, 95% CI = 0.91-1.09, p = 0.074) and age at transplantation (OR = 0.93, 95% CI = 0.87-1.01, p = 0.118), although these did not reach statistical significance.

In multivariate analysis, AKI remained the strongest independent predictor of graft dysfunction (adjusted OR = 41.69, 95% CI = 4.75-365.82, p < 0.001). Recurrent diarrhea was also independently associated with graft dysfunction after adjustment (adjusted OR = 3.62, 95% CI = 1.01-13.04, p = 0.049). In contrast, drug-induced acute diarrhea was no longer significantly associated with graft dysfunction after adjustment (adjusted OR = 1.71, 95% CI = 0.27-11.01, p = 0.572) (Table 3).

**Table 3:** Factors associated with chronic graft dysfunction following gastrointestinal complications. Data are presented as odds ratios with 95% confidence intervals. Univariate and multivariate binary logistic regression analyses were performed. A p-value <0.05 was considered statistically significant.

Variable	Univariate OR	95% CI	p-value	Adjusted OR	95% CI	p-value
Age	1.04	0.91-1.09	0.074	0.93	0.86-1.01	0.082
Age at transplantation	0.93	0.87-1.01	0.118	0.93	0.87-1.00	0.060
Recurrent diarrhea	2.91	1.29-6.55	0.010	3.62	1.01-13.04	0.049
Drug-induced acute diarrhea	6.35	0.84-48.0	0.070	1.71	0.27-11.01	0.572
Occurrence of acute kidney injury	18.75	3.60-97.5	<0.001	41.69	4.75-365.82	<0.001

**Figure 3:**
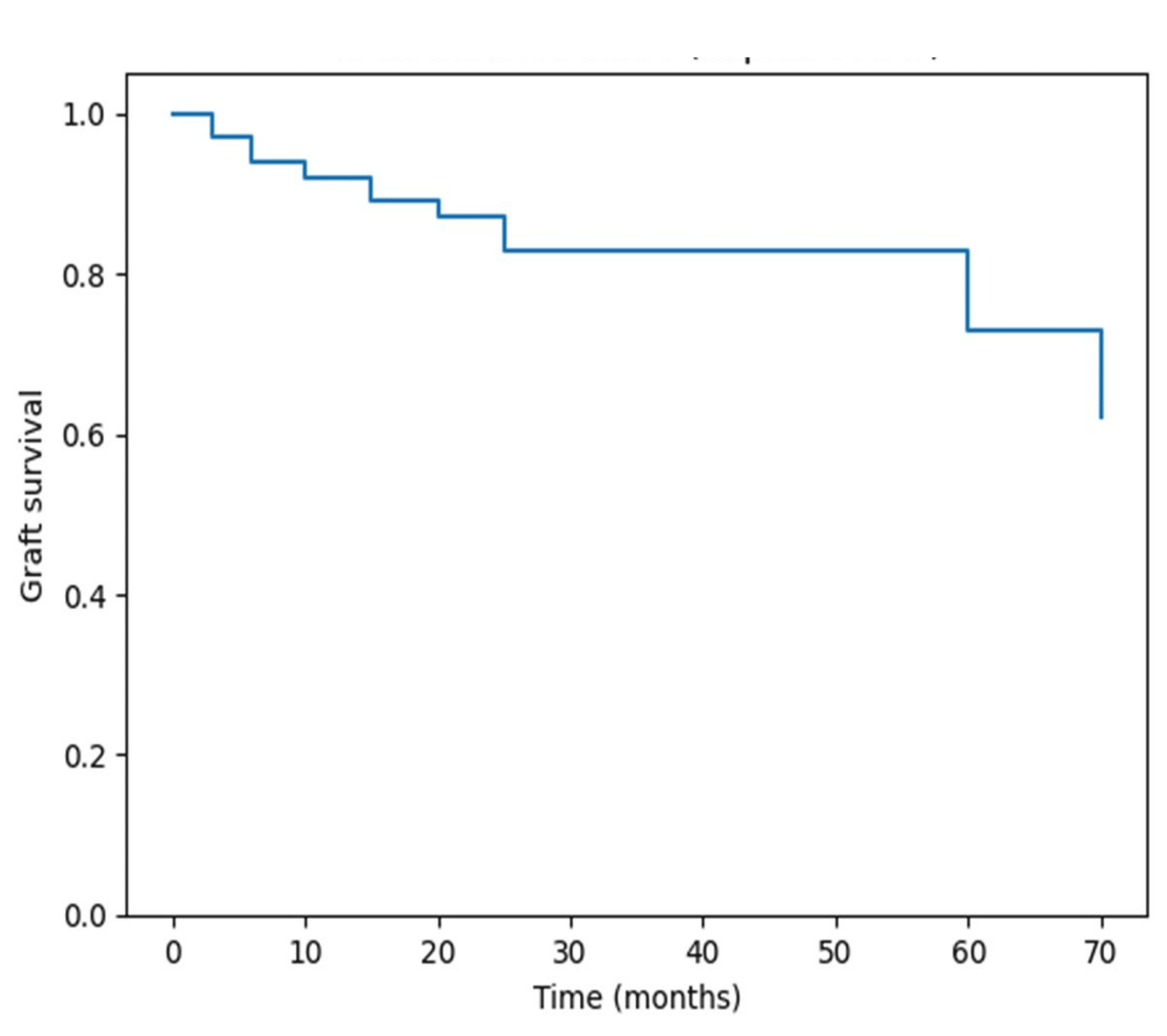
Overall graft dysfunction-free survival curve. Graft survival was estimated using the Kaplan-Meier method and expressed as cumulative survival probability over time (months).

The median graft dysfunction-free survival in our cohort was 60 months (range = 4-70 months). Graft survival was analyzed using the Kaplan-Meier method, considering chronic graft dysfunction as the event of interest. Overall, renal survival declined progressively over time, with a moderate decrease beyond 12 months and a more pronounced decline around 60 months. Despite this trend, a substantial proportion of patients maintained long-term graft function (Figure 3).

**Figure 4:**
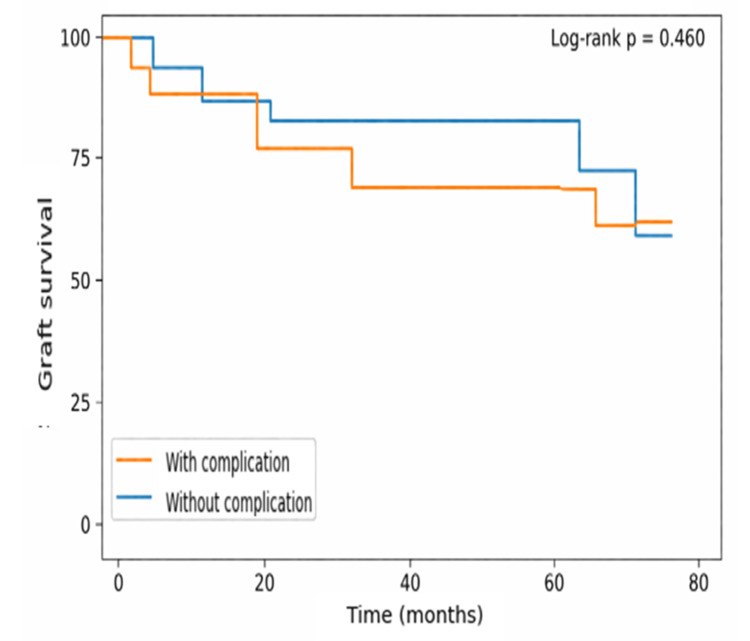
Kidney graft dysfunction-free survival curve based on the presence or absence of gastrointestinal complications following kidney transplantation. Graft survival was estimated using the Kaplan-Meier method and expressed as cumulative survival probability over time (months). Survival curves were compared using the log-rank test. A p-value <0.05 was considered statistically significant.

When stratified by the occurrence of complications, six-year graft dysfunction-free survival appeared comparable between patients with and without complications, with no significant difference observed on Kaplan-Meier analysis (log-rank p = 0.460), although a slight, non-significant trend toward lower survival was noted in patients who developed gastrointestinal events (Figure 4).

**Figure 5:**
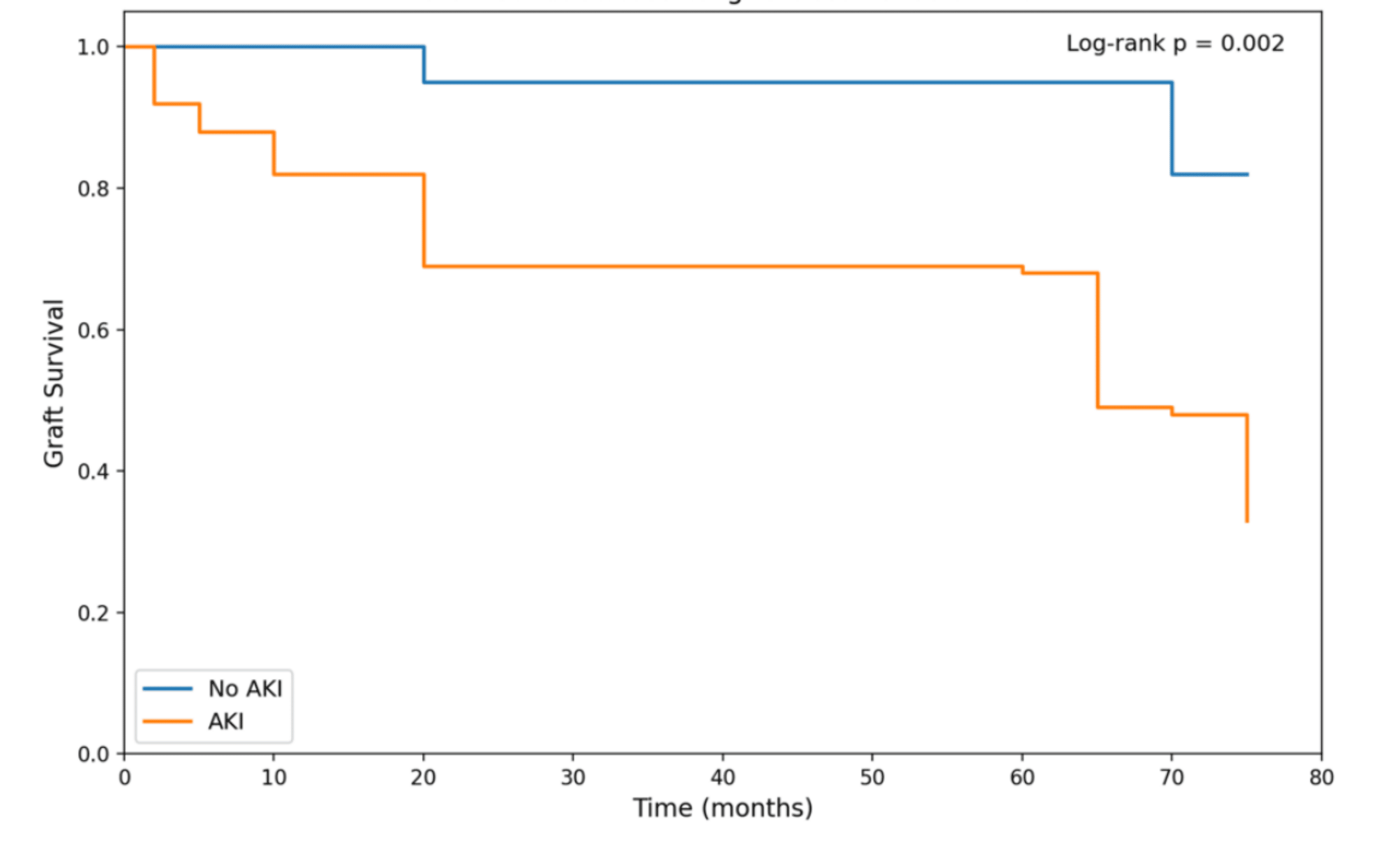
Kidney graft dysfunction-free survival curve based on the presence or absence of gastrointestinal complications-related acute kidney injury (AKI). Graft survival was estimated using the Kaplan-Meier method and expressed as cumulative survival probability over time (months). Survival curves were compared using the log-rank test. A p-value <0.05 was considered statistically significant.

In contrast, graft dysfunction-free survival was markedly reduced in patients who developed AKI, with Kaplan-Meier analysis demonstrating a significant difference between groups (log-rank p = 0.002) (Figure 5).

## Discussion

GICs after KT remain a frequent and clinically significant issue, although their true incidence is difficult to estimate due to heterogeneity in definitions and clinical presentations. Reported rates vary widely across studies, with an incidence of approximately 12.6% in some series [10]. In our study, these complications were particularly common, affecting nearly half of the patients (46.9%), with a total of 84 events analyzed, highlighting a substantial burden in our population.

Diarrhea was by far the most frequent clinical manifestation (85.71%), consistent with previous reports. The MITOS study showed that 53% of kidney transplant recipients experienced at least one GIC, with diarrhea being the predominant presentation [11]. In contrast, large database analyses such as the National Inpatient Sample (NIS) reported a lower incidence (around 5%), likely reflecting differences in case definitions and underreporting of less severe manifestations [10].

The timing of these complications is also crucial. In our series, nearly half of the events (48%) occurred during the first year following transplantation, a period recognized as high risk due to intense immunosuppression, exposure to potentially toxic therapies, and increased susceptibility to opportunistic infections. This observation is consistent with previous studies showing that approximately half of major GICs arise during the first post-transplant year, particularly surgical complications, postoperative ileus, gastroduodenal ulcers, and early infections [12-14].

Infectious etiologies were predominant in our cohort, with viral infections leading the spectrum. SARS-CoV-2 (COVID-19) was the most frequent cause, followed by CMV, both representing major contributors to digestive morbidity. These findings are supported by the literature: Kates et al. reported gastrointestinal manifestations in 47.9% of transplant recipients with COVID-19, predominantly diarrhea [15], while Cravedi et al., from the TANGO International Transplant Consortium, found gastrointestinal involvement in 38% of kidney transplant recipients, sometimes as the initial presentation [16].

In addition to infections, immunosuppressive therapy plays a central role in the development of GICs. MMF and tacrolimus are the most frequently implicated agents, particularly when used in combination. The MMF-tacrolimus regimen has been associated with a higher incidence of gastrointestinal symptoms, especially diarrhea [11]. MMF and its active metabolite, mycophenolic acid, are well known to induce dose-dependent gastrointestinal toxicity, affecting the entire digestive tract [17]. Furthermore, even independently of MMF, tacrolimus has been associated with a high burden of digestive symptoms, as demonstrated in a randomized controlled trial by Al Fatly et al. [18].

Beyond their frequency, these complications have a significant impact on graft outcomes. Gastrointestinal manifestations, particularly diarrhea, are associated with both acute and chronic deterioration of renal function. In this context, Kim et al. reported that AKI occurred in 53.6% of patients with GICs and was strongly associated with reduced graft survival [19]. Survival analysis further demonstrated that patients without diarrhea had the best graft outcomes, whereas those with diarrhea complicated by AKI had significantly worse survival (log-rank p = 0.049) [19].

These findings suggest that post-transplant diarrhea is not merely a benign complication but a true prognostic factor. Several mechanisms may explain this association, including recurrent dehydration, impaired absorption of immunosuppressive drugs leading to fluctuations in drug levels, and therapeutic adjustments that may increase the risk of acute rejection [19].

Our study has several limitations that should be acknowledged. First, its retrospective single-center design may have introduced selection bias and information bias related to incomplete data collection and missing diagnostic workup.

Second, the long study period, spanning over a decade, represents a particularly important source of temporal bias. Transplant practices, immunosuppressive protocols, and diagnostic strategies evolved substantially over time.

Most notably, COVID-19 emerged as the leading viral etiology of diarrhea in our cohort, which likely reflects pandemic-era ascertainment bias rather than a generalizable post-transplant GIC profile. During the COVID-19 pandemic, kidney transplant recipients, as immunocompromised patients, were particularly susceptible to SARS-CoV-2-related gastrointestinal manifestations, and the heightened clinical awareness and systematic testing for SARS-CoV-2 during this period may have led to its overrepresentation among identified etiologies. This period effect may therefore limit the generalizability of our etiological distribution to non-pandemic settings and should be interpreted with caution.

Third, the relatively small sample size limits the statistical power of the analysis, particularly for subgroup analyses and the assessment of less frequent events, and may have resulted in underpowered estimates for some associations.

These limitations highlight the need for larger prospective multicenter studies with systematic diagnostic workup and stratification by transplantation era to confirm our findings and better characterize the clinical impact of GICs on kidney transplant outcomes.

Overall, our results highlight the multifactorial nature of GICs after KT and their potential impact on graft function and survival. Early recognition, accurate etiological diagnosis, and appropriate management are essential to reduce morbidity and improve long-term transplant outcomes.

## Conclusions

GICs are common after KT and represent a significant clinical concern. Often underestimated, they arise from the interplay between immunosuppressive therapy, infectious risk, and patient-related factors.

In our study, diarrhea emerged as the predominant manifestation and was closely associated with impaired graft function. AKI frequently complicated these events and appeared to be a key determinant of subsequent chronic graft dysfunction, highlighting its prognostic significance. However, these results should be interpreted with caution. Given the retrospective single-center design, small sample size, long study period spanning over two decades, and the exploratory nature of the analysis, causal inference cannot be established. These findings emphasize the need for early diagnosis, careful etiological assessment, and optimized management strategies. Improving the recognition and treatment of GICs may ultimately enhance graft outcomes and patient prognosis in kidney transplant recipients.
